# Transcriptome analysis of desmoplastic small round cell tumors identifies actionable therapeutic targets: a report from the Children’s Oncology Group

**DOI:** 10.1038/s41598-020-69015-w

**Published:** 2020-07-23

**Authors:** Pooja Hingorani, Valentin Dinu, Xiyuan Zhang, Haiyan Lei, Jack F. Shern, Jin Park, Jason Steel, Femina Rauf, David Parham, Julie Gastier-Foster, David Hall, Douglas S. Hawkins, Stephen X. Skapek, Joshua Labaer, Troy A. McEachron

**Affiliations:** 1 UT MD Anderson Cancer Center, 1515 Holcombe Blvd, Houston, TX 77030 USA; 2 The Biodesign Institute, OKED Genomics Core, Arizona State University, Tempe, AZ USA; 3 Pediatric Oncology Branch, National Cancer Institute, Bethesda, MD USA; 4 Department of Pathology, Children’s Hospital of Los Angeles, Los Angeles, CA USA; 5 Institute for Genomic Medicine, Nationwide Children’s Hospital, Columbus, OH USA; 6 Departments of Pathology and Pediatrics, Ohio State University College of Medicine, Columbus, OH USA; 7 Division of Biostatistics, Children’s Oncology Group, Monrovia, CA USA; 8 Division of Pediatric Hematology Oncology, Seattle Children’s Hospital, Seattle, WA USA; 9 University of Washington, Fred Hutchinson Cancer Research Center, Seattle, WA USA; 10 Division of Pediatric Hematology Oncology, UT Southwestern Medical Center, Dallas, TX USA; 11 Department of Translational Genomics, Keck School of Medicine, University of Southern California, Los Angeles, CA USA; 12 Department of Pediatrics, Keck School of Medicine, University of Southern California, Los Angeles, CA USA

**Keywords:** Cancer, Sarcoma

## Abstract

To further understand the molecular pathogenesis of desmoplastic small round cell tumor (DSRCT), a fatal malignancy occurring primarily in adolescent/young adult males, we used next-generation RNA sequencing to investigate the gene expression profiles intrinsic to this disease. RNA from DSRCT specimens obtained from the Children’s Oncology Group was sequenced using the Illumina HiSeq 2000 system and subjected to bioinformatic analyses. Validation and functional studies included WT1 ChIP-seq, EWS-WT1 knockdown using JN-DSRCT-1 cells and immunohistochemistry. A panel of immune signature genes was also evaluated to identify possible immune therapeutic targets. Twelve of 14 tumor samples demonstrated presence of the diagnostic *EWSR1-WT1* translocation and these 12 samples were used for the remainder of the analysis. RNA sequencing confirmed the lack of full-length WT1 in all fusion positive samples as well as the JN-DSRCT-1 cell line. ChIP-seq for WT1 showed significant overlap with genes found to be highly expressed, including *IGF2* and *FGFR4*, which were both highly expressed and targets of the EWS-WT1 fusion protein. In addition, we identified *CD200* and *CD276* as potentially targetable immune checkpoints whose expression is independent of the EWS-WT1 fusion gene in cultured DSCRT cells. In conclusion, we identified *IGF2*, *FGFR4, CD200*, and *CD276* as potential therapeutic targets with clinical relevance for patients with DSRCT.

## Introduction

DSRCT was first described by Gerald and Rosai^1^ as a highly malignant soft-tissue sarcoma occurring in adolescent and young adults, especially in males. It often presents with widespread disease with malignant foci present throughout the peritoneal cavity. Despite intense multi-agent chemotherapy, surgery, radiation therapy, and incorporation of other treatment modalities like high-dose chemotherapy with autologous stem cell rescue, 5-year event-free survival remains dismal at less than 20%, while the median survival time is approximately 2.5 years^2^. Novel therapies are therefore urgently needed to improve the outcomes for patients with this disease.

Like most solid malignancies in this age range, the cell of origin for DSRCT remains elusive, but it is thought to represent a primitive mesenchymal precursor^3^. The hallmark molecular characteristic of DSRCT is a reciprocal t(11;22)(p13;q12) translocation involving the *EWSR1* and the *WT1* genes, identified in 1994^4^. Histologically, DSRCTs appear as nests of “small round blue cells” surrounded by a dense desmoplastic stroma. Immunohistochemical analysis suggests multi-lineage differentiation that includes expression of epithelial, mesenchymal, and neuronal markers^5^.

Most commonly, the t(11;22)(p13;q12) translocation “fuses” exon 7 of *EWSR1* to exon 8 of *WT1*, although variants with different splice sites are described, including fusions between exon 8 of *WT1* to exons 9 or 10 of *EWSR1*^6^. The fusion mRNA transcript encodes the chimeric *EWSR1*-*WT1* transcription factor, expression of which has been reported to regulate platelet derived growth factor alpha (*PDGFA*)^7^, cytokine receptor *IL-2* receptor beta, exocytosis regulator *BAIAP3*, T-cell associated acute lymphoblastic leukemia-associated antigen (*TALLA-1*), myeloid leukemia factor-1 (*MLF-1*), insulin-like growth factor-I receptor (*IGF-IR*), connective tissue growth factor 2 (*CCN*2), and leucine rich containing repeat 15 (*LRRC15)* genes expression levels^8–13^. Although, these studies provide certain insights into the potential oncogenic pathways involved in DSRCT, gaps in our understanding of DSRCT biology hampers our ability to identify and prioritize possible therapeutic targets.

In this study, we try to close this gap using RNA-seq, ChIP-seq, and functional studies to identify targets of potential therapeutic utility.

## Methods

### Patients and samples for RNA-seq analysis

This study was conducted with approval from the Children’s Oncology Group’s (COG) Soft-Tissue Sarcoma Committee to obtain DSRCT specimens collected and archived at the biospecimen bank located at the Biopathology Center (BPC) of Nationwide Children’s Hospital, Columbus, OH under the study protocol ARST16B3-Q. The specimens banked at the BPC had prior informed consents that were obtained from parent/legal guardian at the time of collection to be able to be used for future banking and research. In addition, institutional review board approvals at Phoenix Children’s Hospital were obtained prior to start of the study and all methods were performed in accordance with the relevant guidelines and regulations. Histological confirmation of disease and tumor quality assurance prior to genomic extraction from the samples was provided by the BPC pathologist and subsequent genomic extraction was performed at the BPC. Tumor RNA was available for 14 patient samples. Limited clinical and pathological information on these samples was available from the COG data center under the study protocol ARST11B5.

### RT-PCR analysis

100 ng of RNA was reverse-transcribed using the Superscript-III First Strand cDNA Synthesis kit (Thermo Fisher Scientific) and subsequently amplified with Platinum PCR SuperMix HiFi (Thermo Fisher Scientific) using the primers listed in Supplemental Table 1. Amplicons were resolved on a 1% Tris–Acetate–EDTA (TAE) agarose gel and visualized using Gel-Red.

### Cell culture

The JN-DSRCT-1 cell line was kindly provided by Dr. Sean Lee (Tulane University). While the short tandem repeat profile does not exist for this cell line, we did confirm the presence of the *EWSR1-WT1* translocation and fusion gene expression using next generation sequencing and Sanger sequencing. The cells were grown in a 1:1 mixture of DMEM: F12 (Invitrogen) with 10% tetracycline-free fetal bovine serum (Clontech) and 1X Glutamax (Invitrogen). Silencing of WT1 was achieved by transducing the JN-DSRCT-1 cells with lentiviral particles encoding inducible WT1-shRNA plasmids (Dharmacon, clones V3IHSHEG_6590764 and V3IHSHEG_6197767) in the presence of 5ug/mL polybrene. Stably transduced cells were selected with puromycin (3 µg/mL). WT1 silencing was induced by incubating the cells with 1 µg/mL of doxycycline for 48-h.

### WT1 ChIP-Seq and data analysis

JN-DSRCT-1 cells were grown to ~ 90% confluence in a 10 cm dish. The chromatin was prepared using the Covaris TruChIP kit according to the manufacturer’s protocol. Briefly, cells were fixed in 1% methanol-free paraformaldehyde for 5 min, quenched, and lysed. Nuclei were collected and sonicated on a Covaris M220 focused ultrasonicator. The final fragment size was determined using the TapeStation2200 (Agilent) and ranged from 100 to 300 bp. Equal concentration of crosslinked chromatin was immunoprecipitated with antibodies against RNA polymerase II (#39097; Active Motif) or the C-terminus of WT1 (sc-192; Santa Cruz). Crosslinks were reversed with proteinase K and the DNA purified using AMPure beads (Beckman Coulter). Sequencing libraries were made from the purified DNA using the Kapa Hyper Library Preparation Kit (KAPA Biosystems) and sequenced on a HiSeq2500 (Illumina). Independent biological triplicates of this experiment were performed.

ChIP enriched DNA reads were mapped to reference genome (version hg19) using BWA(3). Duplicate reads were discarded. For Integrative Genomics Viewer (IGV) sample track visualization, coverage density maps (tdf files) were generated by extending reads to the average size and counting the number of reads mapped to each 25 bp window using igvtools (https://www.broadinstitute.org/igv/igvtools). ChIP-seq read density values were normalized per million mapped reads. High-confidence ChIP-seq peaks were called by MACS2 (https://github.com/taoliu/MACS) with the narrow algorithm for TFs. The peaks which overlapped with possible anomalous artifact regions (such as high-mappability regions or satellite repeats) blacklisted by the ENCODE consortium (https://sites.google.com/site/anshulkundaje/projects/blacklists) were removed using BEDTools. Peaks from ChIP-seq data were selected using a stringent p-value (e-7) and described by a peak score (− log_10_ p value). The distribution of peaks (as intronic, intergenic, exonic, etc.) and motif analysis was annotated using HOMER.

### RNA sequencing and data analysis

Libraries were generated, quantified, and sequenced on a HiSeq2000 (Illumina) at the Arizona State University Biodesign Institute Sequencing Core Facility. Briefly, RNA from the tumor samples was reverse transcribed the Ovarion RNA-Seq System (NuGEN). The cDNA shearing was performed using the Covaris M220 system to approximately 300 base pair fragments. Sequencing libraries were generated using the KAPA Library Preparation Kit (KAPA Biosystems) and barcoded with NEXTflex indexed adapters (BioO Scientific). Ligated molecules were purified with AMPure beads (Beckman Coulter). The quality and quantity of each library was determined using a Bioanlyzer (Agilent) and qPCR (KAPA Biosystems).

Total RNA from the JN-DSRCT-1 cell line was isolated (NorgenBiotek) and quality assessed with the TapeStation2200 (Agilent). Sequencing libraries were prepared using the Strand Specific RNA Library Preparation Kit (Agilent) according to the manufacturer’s protocol. Library purification and size selection was performed with AMPure beads (Beckman Coulter). The purified libraries were quantified using the TapeStation2200 (Agilent) and sequenced on the HiSeq2500 platform (Illumina) at the Translational Genomics Research Institute.

The RNA-sequencing data was analyzed using a custom bioinformatics pipeline. Briefly, raw data (.bcl files) were converted to .fastq files and aligned with STAR aligner using human genome build hs37d5 as a reference. Ensembl v74 was used for annotation. Expressed fusion genes were detected with TopHat Fusion. Transcript and gene-level Transcripts Per Million (TPM) were generated with Sailfish v0.6.3 and subsequently log2 transformed. Genes with an average gene-level TPM value < 2 across all samples were excluded. TPM values were further log2 transformed for all downstream analyses. Single sample gene set enrichment analysis (ssGSEA) was performed on all fusion positive DSRCT specimens using the top 5,000 genes with the greatest standard deviation across all specimens. “Hallmark gene sets (H)” and “Gene Ontology gene sets (C5)” were used for this analysis for a total of 1,093 terms. Consensus clustering, hierarchical clustering, and differential expression analyses were performed using Morpheus (https://software.broadinstitute.org/morpheus). Histograms were generated using Prism (Graph Pad). The RNA sequencing data have been deposited at the European Genome-phenome Archive (EGA) database under accession number EGAS00001002770. RNA sequencing metrics are summarized in Supplemental Table 2.

### Gene expression array data mining

Publically available gene expression array data from fusion positive sarcomas (rhabdomyosarcoma, alveolar soft part sarcoma, DSRCT, and Ewing sarcoma)^14^ were analyzed using the R2: Genomics Analysis and Visualization Platform (https://r2.amc.nl). Data from u133a chips were MAS5.0 normalized and used for principal component analysis (PCA) and IGF2 and FGFR4 box plots.

### Western blots

Whole cell lysates were created using radio-immunoprecipitation assay (RIPA) buffer supplemented with protease and phosphatase inhibitors (Thermo Scientific). Conditioned media was collected after 48-h of culture in the presence or absence of doxycycline. The conditioned media was cleared of cellular debris by centrifugation and concentrated using the Pierce protein concentrator PES 3 K MWCO (ThermoFisher). Samples were quantified and equal amounts of protein lysate or conditioned media were separated on 4–12% Bis–Tris gradient gels (Thermo Scientific). The proteins were transferred to polyvinylidene fluoride membranes, blocked with 5% bovine serum albumin, and probed with primary antibodies against the C-terminus of WT1 (sc-192; Santa Cruz), FGFR4 (#8562; Cell Signaling), GAPDH (#5174; Cell Signaling), IGF2 (ab9574; Abcam), CD200 (AF2724; R&D Systems), or B7-H3 (#14058; Cell Signaling). The membranes were incubated with HRP conjugated secondary antibodies (anti-rabbit and anti-goat) and subsequently visualized with ECL reagent (Thermo Scientific). The blots were imaged using the ChemiDoc MP system (Bio-Rad). All blots were performed in biological triplicate experiments and representative images are shown.

### Immunohistochemical analysis

An independent cohort of 12 archived formalin-fixed, paraffin embedded DSRCT specimens that were previously molecularly confirmed to have the EWS-WT1 fusion were retrieved from the Department of Pathology at Children’s Hospital Los Angeles. 5 µm slides were cut and de-paraffinized in xylene, rehydrated in graded ethanol, and subjected to heat-induced antigen retrieval in citrate buffer. Endogenous peroxidase quenching and biotin-blocking were performed prior to incubating the slides with non-specific blocking buffer (2% horse serum). The slides were incubated overnight with the respective primary antibodies. Color development and counterstaining were accomplished using 3,3′-diaminobenzidine and hematoxylin (Vector Laboratories, Burlingame, CA). Primary antibodies, anti-FGFR4 (#8562) and anti-B7-H3 (#14058) were obtained from Cell Signaling Technologies.

## Results

### Patient and histopathological tumor characteristics

Table 1 lists the available clinical and histopathological characteristics of all fourteen samples analyzed by RNA-seq in this study. Median age was 14 years (range 2–18 years). There were nine male and five female patients, and the most common anatomic sites were listed as soft tissue of abdomen/pelvis or peritoneum.

**Table 1:** Patient and tumor characteristics.

Patient ID	Sex	Race	Age at diagnosis (years)	Primary tumor site	Biopsy time point	IHC+	IHC−	Genetics
EVW	Male	White	13.5	Soft tissues of pelvis	Diagnosis	Desmin, cytokeratin, NSE, CD99	Actin, WT1, CAM5.2	NA
IZJ	Male	White	15.8	Peritoneum	Diagnosis	Desmin, cytokeratin, EMA	CD99, myogenin, MyoD1	NA
JVH^a^	Male	White	14.8	Soft tissues of pelvis	Diagnosis	Desmin, cytokeratin, CD99, Vimentin, WT1	Myo D1, S100, Actin, EMA, AFP, HMB-45, CD1a, CD21, CD30,CD68	No EWS gene rearrangement by FISH; no sarcoma translocations by RT-PCR
NVF	Female	Unknown	11.9	Abdomen, abdominal wall	Diagnosis	NA	NA	NA
YYR	Male	White	14.7	Soft tissues of pelvis, buttock, groin	Diagnosis	Desmin, vimentin, CD99, NSE, EMA	CD45, myogenin, actin, chromogranin, WT1	EWS-WT1 +
ZDI	Female	White	17.3	Soft tissues of pelvis, buttock, groin	Diagnosis	Desmin, vimentin, NSE, SMA	EMA, WT-1, S-100	EWS-WT1 +
SAW	Female	African American	2	Lymph node, NOS	Diagnosis	Desmin, vimentin, NSE, Synaptophysin, CD99	Myogenin, cytokeratin, EMA	EWS gene rearrangement by FISH
DPZ	Female	White	12.5	Abdomen, abdominal wall	Diagnosis	Desmin, cytokeratin, CD99, NSE	WT-1, CD45	NA
590	Male	NA	15	Abdomen, peritoneal implant, NOS	Diagnosis	Desmin, cytokeratin, EMA, NSE	CD45, CD99, actin	EWS-WT1 +
055	Male	White	10	Abdomen, NOS	Diagnosis	Desmin, pan-keratin, EMA, vimentin	CD99, chromogranin, S-100, O13	NA
056	Male	NA	18	Abdomen, Liver	Diagnosis	Desmin, NSE, cytokeratin, EMA, Vimentin, CD99	O13, LCA	NA
057	Female	NA	10	Peritoneum pelvis	Follow up	NA	NA	NA
059^a^	Male	NA	13	Gastric ulcer	Diagnosis	Cytokeratin, vimentin	Chromogranin, CEA, CD15, AFP, PLAP	NA
060	Male	NA	16	Sigmoid colon, omentum	Diagnosis	NA	NA	NA

*NA* not available, *IHC* immunohistochemistry.

^a^Samples negative for EWS-WT1.

### EWSR1-WT1 analysis in DSRCT samples

**Figure 1:**
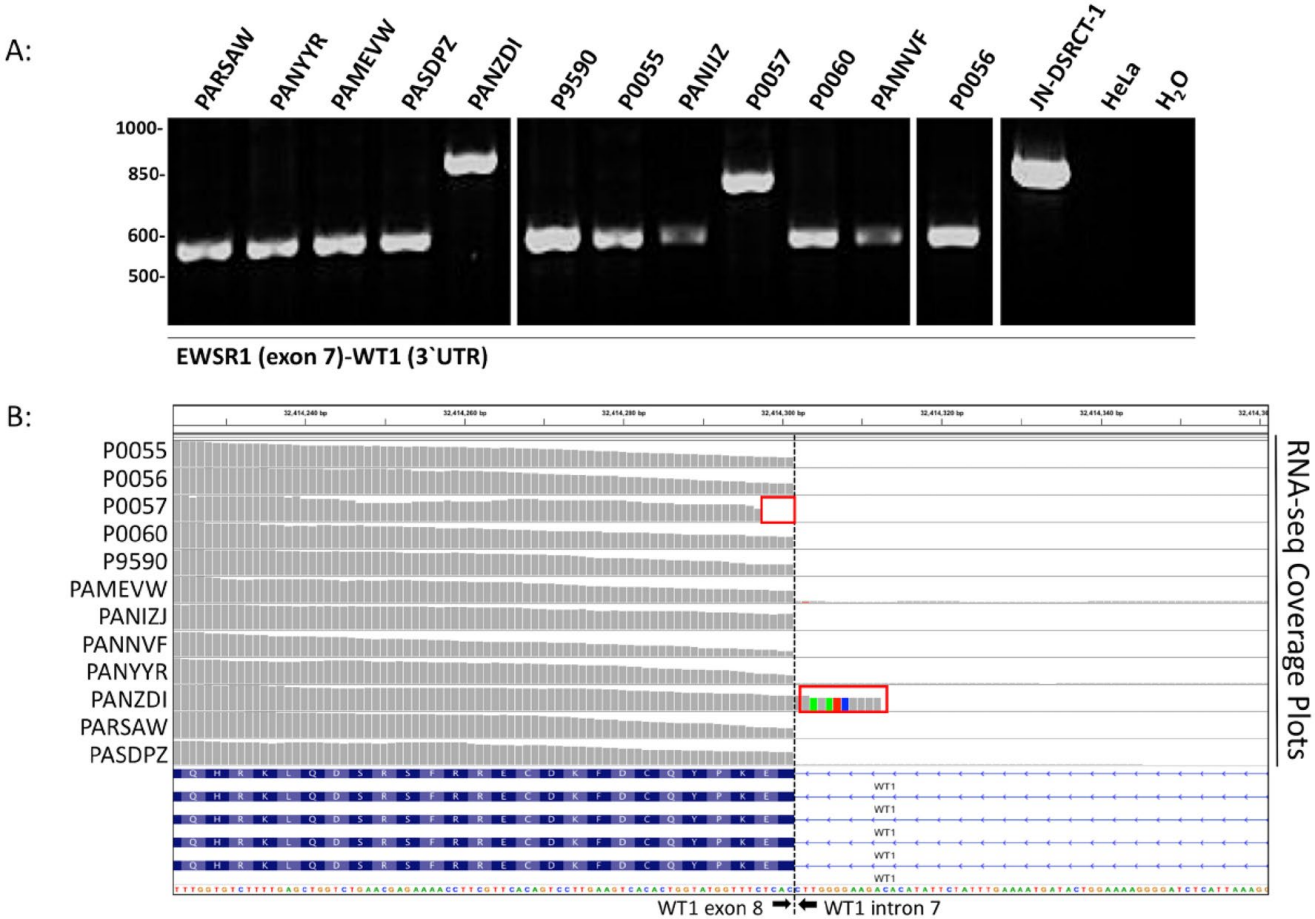
Molecular validation of the *EWSR1*-*WT1* fusion status in the study samples. (**A**) PCR amplification of the *EWSR1-WT1* fusion gene in DSRCT samples using primers that flank the presumptive fusion junction. The JN-DSRCT-1 cell line serves as the positive control for the *EWSR1-WT1* amplicon, HELA cells are the negative control. (**B**) RNA-sequencing reveals splice-site alterations in DSRCT samples expressing alternative *EWSR1-WT1* fusion transcripts. Altered splice sites (red boxes) flanking the intron7/exon8 boundary (black dotted line).

RNA sequencing analysis revealed that two of the fourteen tumors, JVH and 059, were negative for the characteristic *EWSR1-WT1* fusion gene. Reverse transcription PCR using primer combinations (Supplemental Table 1) flanking the presumptive *EWSR1-WT1* junction was performed to validate the expression of the fusion transcript. Agarose gel electrophoresis of the amplicons confirmed the expression of the *EWSR1-WT1* fusion transcript in 12 of the 14 samples (Fig. 1A). All subsequent analysis included only confirmed fusion positive tumors. Two fusion positive samples, ZDI and 057, displayed alternative higher molecular weight amplicons, suggesting the presence of different *EWSR1-WT1* fusion isoforms. Continued analysis of the RNA-sequencing data revealed altered splice sites in these two samples expressing alterative *EWSR1-WT1* fusions (Fig. 1B).

### Clustering analysis of DSRCT specimens

**Figure 2:**
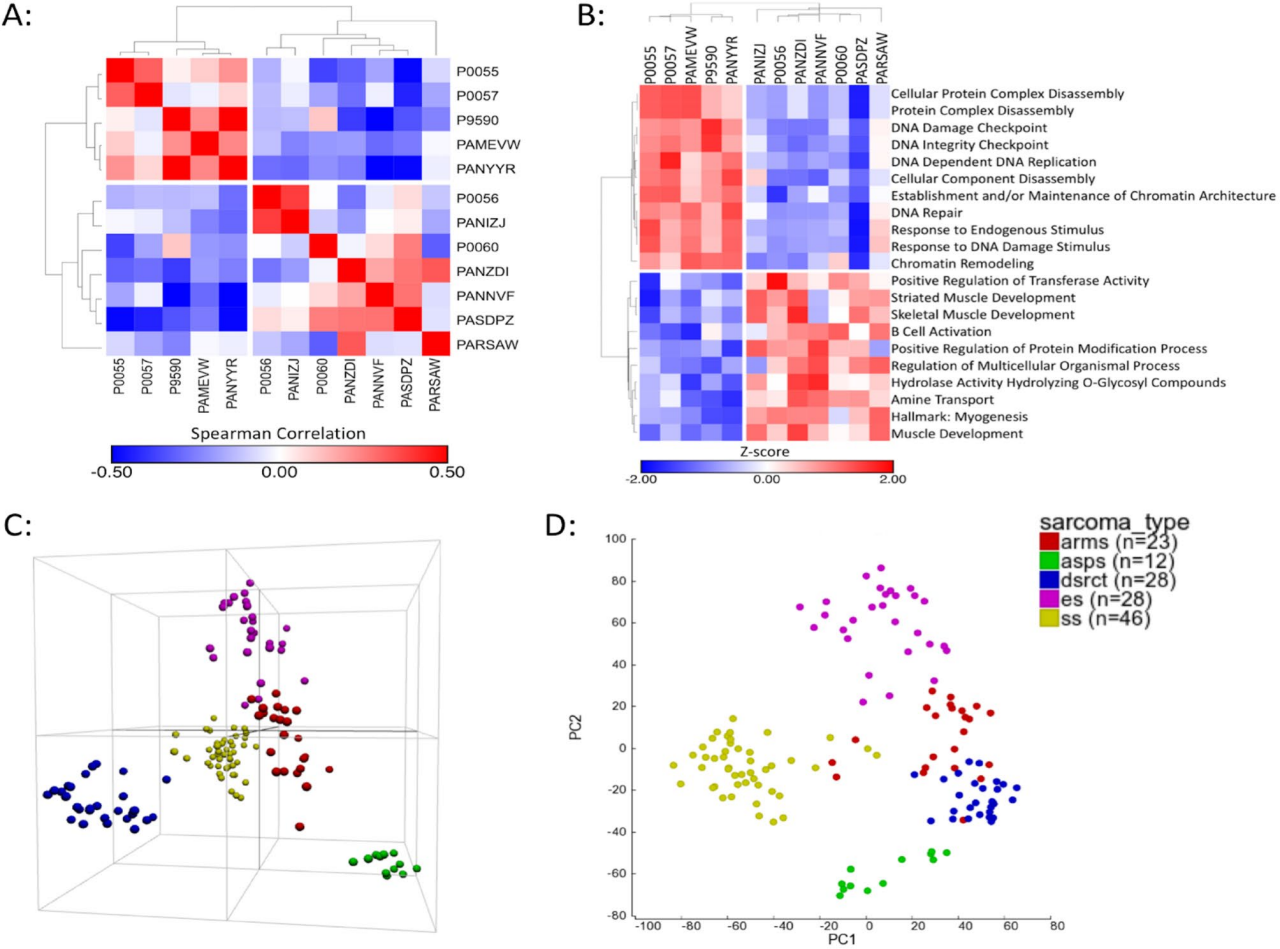
Clustering analysis of DSRCT specimens. (**A**) Consensus clustering and Spearman rank correlation analysis of the DSRCT specimens after ssGSEA analysis demonstrating that DSRCT specimens segregate into two groups. Color bar indicates correlation strength (blue = weaker, red = stronger). (**B**) Unsupervised hierarchical clustering using average linkage and Spearman correlation illustrating the 20 most differentially expressed gene set enrichment terms between the two subgroups of DSRCT patient specimens. Color bar indicates row Z-score. (**C**, **D**) Principal component analysis of gene expression data from alveolar rhabdomyosarcoma (ARMS; red), alveolar softpart sarcoma (ASPS; green), DSRCT (blue), Ewing sarcoma (ES; purple), and synovial sarcoma (SS; gold) displayed as (**C**) 3-dimensional and (**D**) 2-dimensional projections.

Next, we sought to determine if the altered splicing observed in samples ZDI and 057 were associated with a distinct transcriptional profile. ssGSEA and subsequent consensus clustering revealed that the DSRCT specimens segregate into two separate clusters (Fig. 2A). Additionally, specimens ZDI and 057 occupy different clusters indicating that the alterative splicing does not confer distinct transcriptional profiles. Analysis of the ten most enriched gene sets in each cluster shows that, collectively, DNA damage response and muscle development are the most significantly differentially enriched gene sets in the two DSRCT clusters (Fig. 2B; Table 2). Using publically available gene expression data, we evaluated the relationship between DSRCT and other fusion positive sarcomas that arise in the adolescent/young adult age range. PCA shows that DSRCT specimens cluster independently of alveolar soft part sarcoma, Ewing sarcoma, alveolar rhabdomyosarcoma, and synovial sarcoma specimens (Fig. 2C,D).

**Table 2:** Significant differentially enriched gene sets in DSRCT.

GSEA term	p value	FDR (BH)
Cellular protein complex disassembly	< 0.001	0.03
Protein complex disassembly	< 0.001	0.03
DNA damage checkpoint	< 0.001	0.02
DNA integrity checkpoint	< 0.001	0.02
DNA dependent DNA replication	< 0.001	0.03
Cellular component disassembly	< 0.001	0.03
Establishment and or maintenance of chromatin architecture	< 0.001	0.03
DNA repair	< 0.001	0.03
Response to endogenous stimulus	< 0.001	0.03
Response to DNA damage stimulus	< 0.001	0.03
Chromatin remodeling	< 0.001	0.03
Positive regulation of transferase activity	< 0.001	0.05
Striated muscle development	< 0.001	0.06
Skeletal muscle development	< 0.001	0.05
B cell activation	< 0.001	0.07
Positive regulation of protein modifcation process	< 0.001	0.04
Regulation of multicellular organismal process	< 0.001	0.05
Hydrolase activity hydrolyzing O-glycosyl compounds	< 0.001	0.05
Amine transport	< 0.001	0.05
Hallmark: myogenesis	< 0.001	0.02
Muscle development	< 0.001	0.02

*GSEA* Gene Set Enrichment Analysis, *FDR (BH)* False Discovery Rate corrected using the Benjamini–Hochberg procedure.

### WT1 ChIP-seq correlates with EWS-WT1 gene signature in JN-DSRCT-1 cells and primary tumor tissues

**Figure 3:**
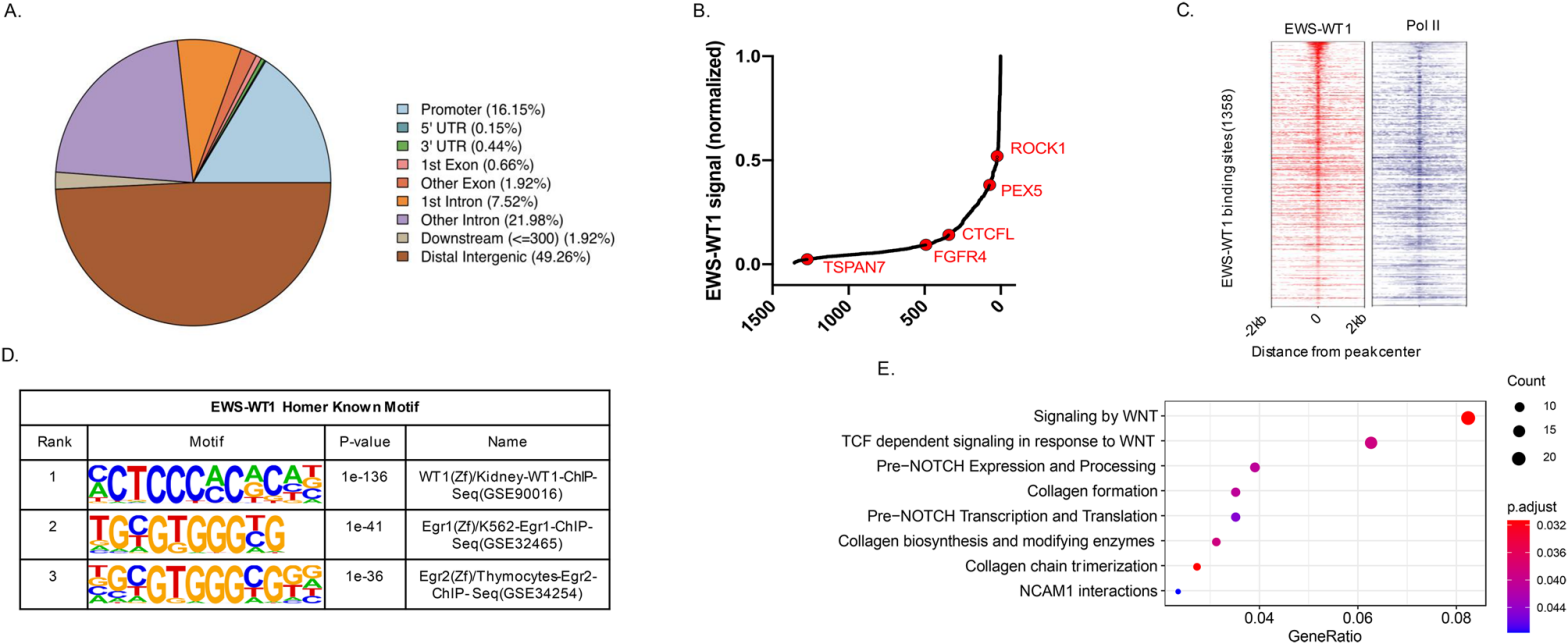
EWS-WT1 ChIP-seq using the JN-DSRCT-1 cell line. (**A**) 2036 Peaks were found to be significantly enriched and a majority of the peaks were found within the intergenic and intronic regions of the genome. (**B**) 1,284 peaks were associated with a protein-coding gene including *ROCK1, PEX5*, *CTCFL*, *FGFR4*, *IGF2* and *TSPAN7.* (**C**) Co-occupancy analysis of EWS-WT1 with RNA polymerase. (**D**) Analysis of the motifs associated with EWS-WT1 peaks showed enrichment for known WT1 binding sequence. (**E**) Pathway analysis demonstrated enrichment in several potentially targetable pathways.

To define the relationship between the EWS-WT1 transcription factor genomic occupancy sites and the observed gene expression profile of the DSRCT tumors, ChIP-seq was performed using an antibody specific for the C-terminus of WT1 in the JN-DSRCT-1 cell line. It has been previously published that most *EWS-WT1* fusion proteins retain the *WT1* zinc finger domains 2, 3, and 4 and demonstrate similar DNA binding specificity for response elements recognized by *WT1*^15^. We were unable to detect the expression of wild-type WT1 in the 12 fusion-positive DSRCT specimens or in the JN-DSRCT-1 cell line as demonstrated by the sashimi plots of the RNA-seq data (Supplemental Fig. 1). WT1 immunoprecipitation and western blotting demonstrated that the αWT1 antibody identifies a single band and that the intensity of the identified band is diminished upon silencing of WT1 (Supplemental Fig. 2). Together, these data indicate that the anti-WT1 antibody used for ChIP-seq bound to the EWS-WT1 fusion protein as intended. The genomic peaks associated with enrichment in sequencing reads vs. control are described in Supplementary Table 3. In total, 2036 statistically significant peaks were discovered of which 1,284 were associated with a protein coding gene. The WT1 peaks showed a marked enrichment for intergenic and intronic genomic locations (Fig. 3A) consistent with other sarcoma fusion oncogenes’ occupancy at genomic enhancers^16,17^. Statistically significant enrichment peaks (Fig. 3B) were found within genes encoding known (*TSPAN7*)^10^ and novel putative targets of the fusion oncogene such as *IGF2*, *FGFR4*, *CTCFL*, *PEX5* and *ROCK1*. ChIP sequencing of RNA polymerase from the same cell line showed frequent co-occupancy of EWS-WT1 suggesting presence of the fusion at actively transcribed genes (Fig. 3C). Motif analysis identified significant enrichment of WT1 binding sequence under the identified peaks (Fig. 3D). Pathway analysis of the genes associated with the discovered ChIP-seq peaks showed an enrichment for genes involved in multiple potentially targetable pathways including, Wnt Signaling, Notch signaling and components of the extracellular matrix (Fig. 3E).

### IGF2 is a highly expressed direct target of the EWSR1-WT1 fusion

**Figure 4:**
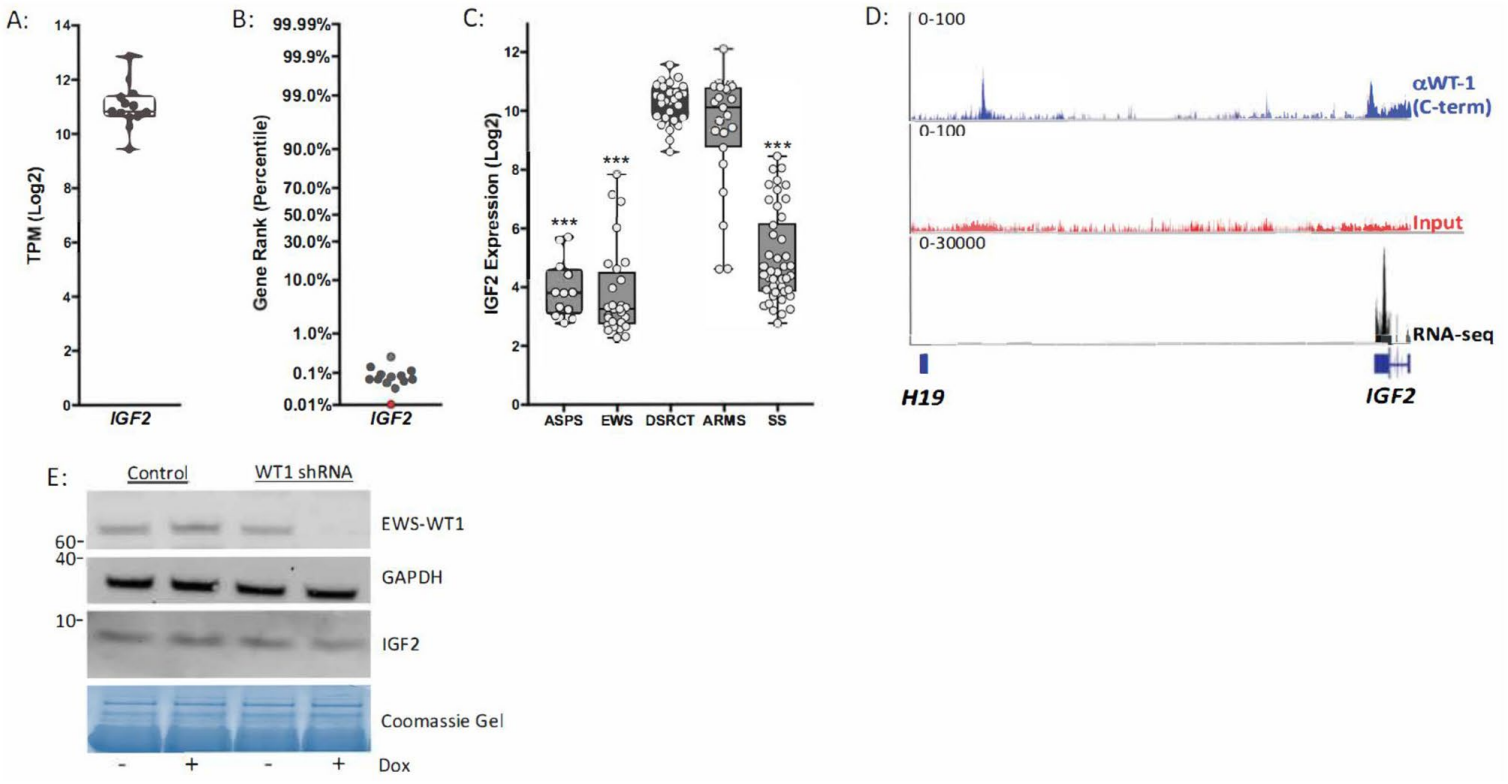
*IGF2* is a highly expressed direct target of EWS-WT1 in DSRCT cells. (**A**) *IGF2* gene expression in patient samples (gray). (**B**) Characterization of *IGF2* expression in patient samples (gray) and the JN-DSRCT-1 cell line (red) using ranked gene list of all genes with TPM (log2) values > 2. (**C**) Comparative analysis of *IGF2* expression across multiple fusion-positive pediatric sarcomas using gene expression array data. *ASPS* (alveolar soft part sarcoma), *EWS* (ewing sarcoma), *DSRCT* (desmoplastic small round cell tumor), *ARMS* (alveolar rhabdomyosarcoma), *SS* (synovial sarcoma). (**D**) EWS-WT1 (blue), input control (red), ChIP-seq peaks and RNA-seq coverage (black) corresponding to the *IGF2* (right) and *H19* (left) genomic loci. (**E**) Western blot of EWS-WT1, GAPDH, and IGF2 (conditioned media) protein expression in JN-DSRCT cells transduced with inducible control shRNA or inducible WT1 shRNA with and without 48-h doxycycline treatment. Independent biological triplicates of the western blotting experiment were performed and a single representative example is shown.

Rank-ordered analysis of the RNA-sequencing data demonstrated robust expression of *IGF2* across all of the confirmed fusion-positive DSRCT samples as well as in the JN-DSRCT-1 cell line (Fig. 4A), where it ranks within the top 0.5% of all genes expressed (Fig. 4B). Using mined gene expression array data^14^, we compared the expression of *IGF2* in DSRCT to that of other fusion positive sarcomas. This analysis revealed that *IGF2* is consistently highly expressed in DSRCT (Fig. 4C). Furthermore, with the exception of fusion-positive alveolar rhabdomyosarcomas, *IGF2* expression in DSRCT is significantly higher than in other fusion positive sarcomas. ChIP-seq using an antibody against WT1 was performed to identify actively transcribed EWS-WT1 target genes and this identified peaks at the *IGF2* genomic locus, demonstrating that EWS-WT1 directly targets *IGF2* (Fig. 4D). However, silencing the expression of the EWS-WT1 fusion using indicuble shRNA did not substantially decrease the amount of IGF2 released from the JN-DSRCT-1 cells in vitro (Fig. 4E). Uniparental disomy of the paternal allele at the 11p15.5 locus results in increased IGF2 expression due to loss of imprinting^18^. Interestingly, we also observed ChIP-seq peaks corresponding to the *H19* differentially methylated region (H19-DMR) located between the *IGF2* and *H19* loci at 11p15.5 (Fig. 4D).

### *Expression of FGFR4 in DSRCT *in vivo* and *in vitro

**Figure 5:**
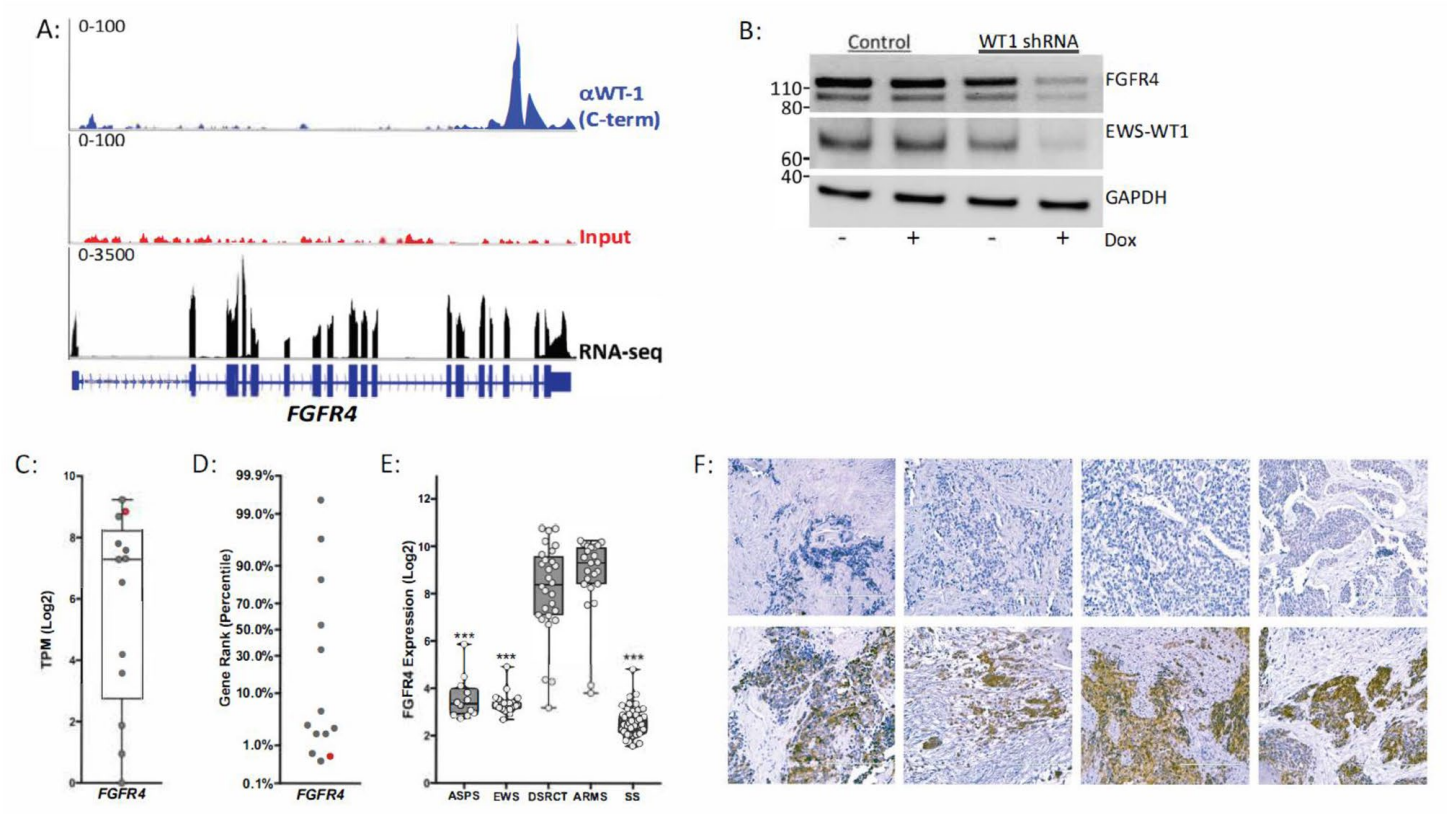
EWS-WT1 regulates FGFR4 expression in DSRCT cells and exhibits variable expression in patient samples. (**A**) EWS-WT1 (blue), input control (red), ChIP-seq peaks and RNA-seq coverage (black) corresponding to the *FGFR4* genomic locus. (**B**) Western blot of FGFR4, EWS-WT1, and GAPDH protein levels in JN-DSRCT cells transduced with inducible control shRNA or inducible WT1 shRNA with and without 48-h doxycycline treatment. Independent biological triplicates of the western blotting experiment were performed and a single representative example is shown. (**C**) *FGFR4* gene expression in patient samples (gray) and the JN-DSRCT-1 cell line (red). (**D**) Characterization of *FGFR4* expression in patient samples (gray) and the JN-DSRCT-1 cell line (red) using ranked gene list of all genes with TPM (log2) values > 2. (**E**) Comparative analysis of *FGFR4* expression across multiple fusion-positive pediatric sarcomas using gene expression array data. (**F**) Low level (top panel) and high level (bottom panel) expression of FGFR4 in DSRCT patient tissues via immunohistochemistry (representative images); each panel represents a different patient.

ChIP-seq data established that EWS-WT1 binds the *FGFR4* locus in JN-DSRCT-1 cells (Fig. 5A). The regulatory role of EWS-WT1 on *FGFR4* expression was further investigated using JN-DSRCT-1 cells stably expressing an inducible shRNA against the 3′ end of WT1. Silencing EWS-WT1 expression in JN-DSRCT-1 cells resulted in decreased expression of the fusion oncoprotein as well as a decrease in FGFR4 protein expression (Fig. 5B). While *FGFR4* was highly expressed in the JN-DSRCT-1 cell line, we observed variable expression in the tumor samples (Fig. 5C). Characterizing *FGFR4* expression using a rank-ordered gene list revealed that DSRCT samples can be distinctly subcategorized into either FGFR4-high or FGFR4-low groups (Fig. 5D). Using the same mined gene expression dataset as above^14^, the expression levels of *FGFR4* were noted to be statistically higher in DSRCT than in other fusion positive sarcomas with the exception of fusion-positive alveolar rhabdomyosarcoma (Fig. 5E). Immunohistochemical analysis also demonstrated that FGFR4 expression is variable in molecularly confirmed DSRCT, thus confirming the RNA sequencing and gene expression array data (Fig. 5F).

### Immunotherapeutic targets CD200 and B7H3 are expressed in DSRCT, independent of EWS-WT1

**Figure 6:**
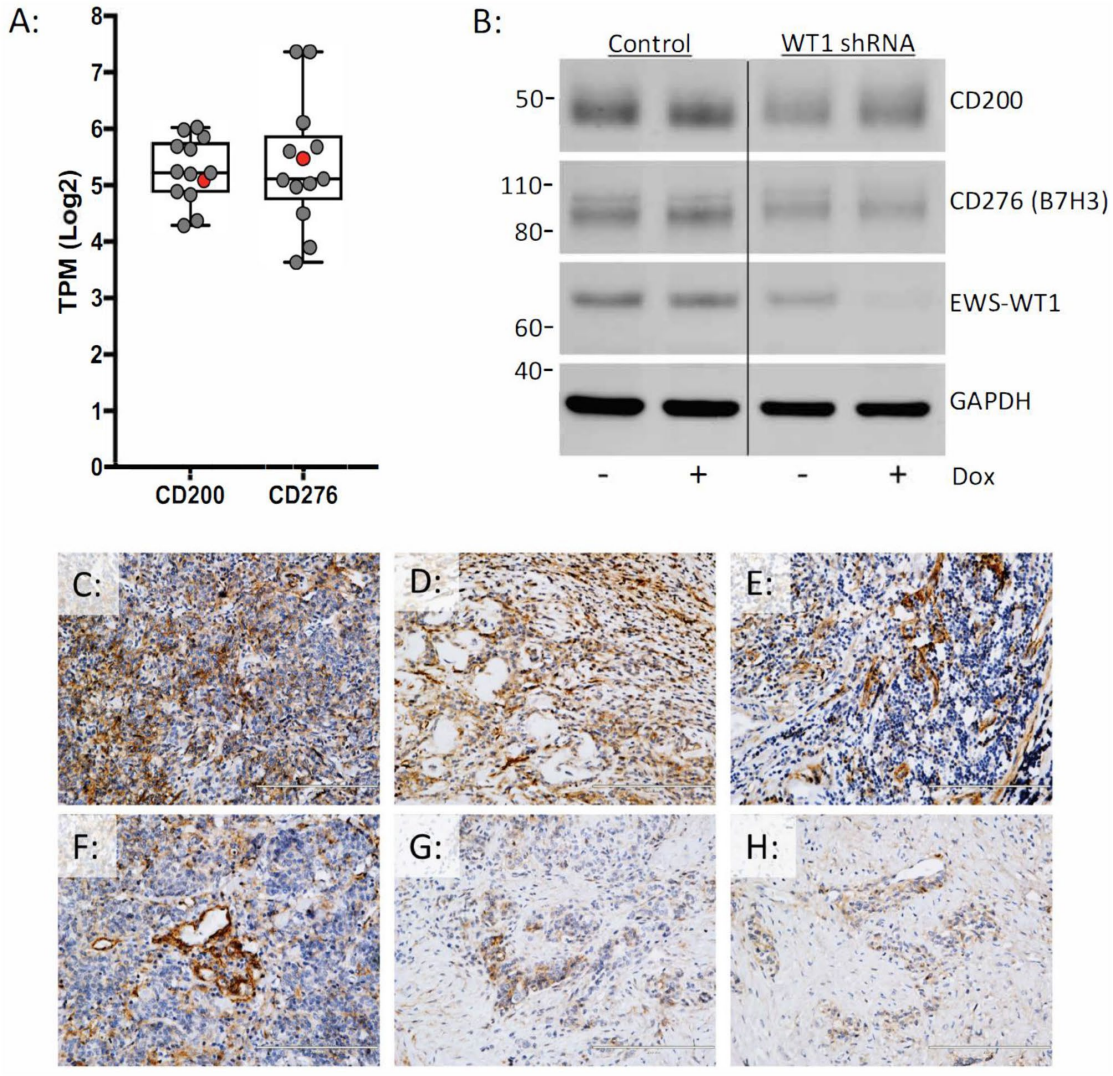
DSRCTs express CD276/B7H3 and CD200 in a EWS-WT1 independent manner. (**A**) *CD200* and *CD276/B7H3* gene expression in patient samples (gray) and the JN-DSRCT-1 cell line (red). (**B**) Western blot of CD200, CD276/B7H3, EWS-WT1, and GAPDH protein levels in JN-DSRCT cells transduced with inducible control shRNA or inducible WT1 shRNA with and without 48-h doxycycline treatment. Independent biological triplicates of the western blotting experiment were performed and a single representative example is shown. (**C**–**H**) Immunohistochemical analysis of CD276/B7H3 expression in DSRCT patient samples (representative images); each panel represents a different patient.

We analyzed the RNA-sequencing data to investigate the targetable immune checkpoint expression profile in DSRCT. Of the genes queried, *CD276* (B7-H3) and *CD200* had the highest expression in both the tumor samples and the JN-DSRCT-1 cell line (Fig. 6A). Silencing EWS-WT1 did not have any measureable effect on the expression of B7-H3 or CD200 protein levels in vitro (Fig. 6B). Protein expression of B7-H3/CD276 was confirmed using immunohistochemistry (Fig. 6C,D). Immunohistochemical analysis demonstrated positive staining within the nests of tumor cells. The staining pattern was not observed in immune infiltrates (Fig. 6C). Despite numerous attempts, we were unable to optimize the conditions for CD200 immunohistochemistry.

## Discussion

DSRCT is a malignancy primarily driven by the EWS-WT1 fusion transcript which in turn upregulates or downregulates downstream genetic targets that impact several inter-connected cancer pathways responsible for oncogenesis. Our study highlights the importance of defining DSRCTs by its hallmark translocation as the two tumor samples in our cohort that were negative for the EWS-WT1 fusion but were diagnosed as DSRCT histologically, had clearly distinct gene expression profiles as compared to the fusion positive tumors (data not shown). Unfortunately, we did not have adequate clinical information on these patients to determine if their clinical presentation and outcome were similar to fusion positive DSRCT patients. Our data goes on to show that DSRCT cluster independently of other fusion positive sarcomas that arise in patients within the same age range.

Our study also shows that *IGF2* is among the most highly expressed genes in both molecularly confirmed DSRCT tissues as well as an established DSRCT cell line. ChIP-seq experiments demonstrated that *IGF2* is a direct target of EWS-WT1 yet that silencing EWS-WT1 alone is not sufficient to decrease the expression of IGF2 in vitro. These data suggest that EWS-WT1 may regulate the expression of IGF2 as part of a larger transcriptional regulatory complex. Previous literature has suggested that EWS-WT1 (− KTS) isoform targets the promoter of *IGF1R*^19^. Our ChIP-seq experiments did not demonstrate a statistically significant enrichment at the *IGF1R* locus. The discrepancy in these results may be due to the fact that the JN-DSRCT-1 cell line used in our investigation expresses both the (− KTS) and (+ KTS) EWS-WT1 isoforms at equivalent levels (Supplemental Fig. 2). It has been reported that the EWS-WT1 (− KTS) and EWS-WT1 (+ KTS) fusion oncoproteins induce distinct transcriptional profiles upon ectopic expression, suggesting that either each fusion targets different genes or that the fusions have opposing regulatory functions^20^. Expression of both isoforms was also observed in DSRCT patient tumor samples (Supplemental Fig. 2), highlighting the complex gene regulatory mechanisms that drive this enigmatic disease. Molecular dissection of these mechanisms is needed to truly understand the myriad of functions of the EWS-WT1 fusion in DSRCT.

Of clinical significance, IGF2 is one of the ligands of the IGF-1R pathway that leads to cell proliferation and survival and anecdotal evidence of activity of IGF-1R antibody, ganitumab, in DSRCT patients exists^21^. Transgenic animals over-expressing *IGF2* have been shown to be at increased risk of developing mammary gland adenocarcinoma and lung cancer. In humans, dysregulation of *IGF2* is evident in a variety of tumors such as breast cancer, ovarian cancer, Wilm’s tumor and mesenchymal malignancies such as Ewing sarcoma^22^. In addition, epigenetic events such as loss of differential methylation of the imprinting control region 1 (ICR 1) at chromosome 11q15 and other imprinting disorders are considered to be important in cancer development. For example, uniparental disomy of the paternal ICR1 at 11p15, encoding the *IGF2-H19* locus is seen in patients with Beckwith-Weidemann syndrome, which is a known cancer predisposition syndrome^18^. The *IGF2-H19* imprinted loci have also been implicated in other malignancies such as lung cancer, squamous cell carcinoma of the head and neck and esophagus, and colorectal cancer^23–26^. Based on our ChIP-seq data (Fig. 3D), we can hypothesize that there is an epigenetic dysregulation mechanism involving *IGF2/H19* locus that may be involved in the pathogenesis of DSRCTs. Clinically, some IGF2 inhibitors such as MEDI-573 and BI 836845 are in trial development and our data suggests that the IGF2/IGF-1R pathway possibly in combination with other modalities of treatment is a rational therapeutic approach in DSRCTs.

Our data also highlights the potential role of FGFR4 in DSRCT pathogenesis. FGFR4 has been implicated in other pediatric malignancies such as rhabdomyosarcoma as well as adult cancers^27,28^. EWS-WT1 protein has previously been shown to be a potent transactivator FGFR4 in DSRCT. Our data further confirms *FGFR4* to be a downstream target of EWS-WT1. Interestingly, while overall *FGFR4* expression was high in DSRCT tumors, there was significant variability in expression noted both by RNA sequencing as well as immunohistochemistry. The clinical significance of this finding remains to be determined and future studies will be required. Both pan-FGFR and specific FGFR4 inhibitors are in clinical trials for cancer (e.g. NCT02325739; NCT02706691) and would once again be agents worthy of testing in DSRCT patients that express high levels of FGFR4 as part of a combination therapy.

In the era of immune therapies emerging as promising approaches in a variety of malignancies, we specifically wanted to investigate whether there were immune markers/signatures that emerged in our data set as potential therapeutic targets. A recent study showed that 80% (9/11) of DSRCT tumors had some PD-1 expression on tumor cells but not lymphocytes, 18% (2/11) had PD-L1 expression on tumor cells and about 63% (7/11) tumors had some CD8 T cell infiltration^29^. We did not find increased PD-L1 gene expression in our data set. However, we identified B7-H3/CD276 and CD200 as the most highly expressed immune markers in DSRCTs. B7-H3 is a member of the B7 superfamily and considered as a co-inhibitory molecule aiding in T-cell inhibition. B7-H3 expression is seen in a variety of malignancies and a recent meta-analysis suggests that increased expression of B7-H3 may be associated with poor overall and event-free survival^30^. Inhibition of B7-H3 has been shown to inhibit tumor growth and reverse chemotherapy resistance in preclinical studies^31,32^. Antibodies against B7-H3 such as enoblituzumab are in adult and pediatric cancer trials alone and in combination with other checkpoint inhibitors (e.g. NCT02982941; NCT02475213). The observed cell-autonomous expression of B7-H3 in DSRCT may also prove useful as an antigen for CAR-T based therapies.

CD200 is a ubiquitously expressed glycoprotein of the immunoglobulin superfamily and is believed to play a role in self-recognition. The expression of CD200R is restricted predominantly to cells of macrophage and myeloid lineage. CD200-CD200R pathway in the context of tumors has been most extensively studied in hematologic malignancies such as mature B-cell neoplasms, CLL and AML^33^. In solid tumors, CD200 expression has been demonstrated on ovarian cancer, melanoma, neuroblastoma and renal cancer cell lines^34^. This pathway has also been implicated in causing immune-suppression in preclinical studies of brain tumors and blockade of the pathway showed augmented anti-tumor immunity^35,36^. The clinical utility of samalizumab, an anti-CD200 monoclonal antibody, is currently in being investigated in an open label phase Ib/II clinical trial to evaluate its therapeutic efficacy in newly diagnosed acute myeloid leukemia (NCT03013998). The role of this pathway in sarcomas is unknown at this time and not previously described in DSRCTs. Our finding of increased expression of CD200 in DSRCT tumors warrants further detailed investigation of this pathway as a potential therapeutic strategy in this disease and will be the focus of future studies.

In summary, our study of DSRCTs identified several important therapeutically actionable genes/proteins that warrant further investigation of agents that target these, in preclinical and clinical trials, to confirm efficacy of these agents in this disease.

## Supplementary information

Supplementary Information 1.

Supplementary Table 2.

Supplementary Table 3.
