# Supplementary material for: Transcriptome analysis of desmoplastic small round cell tumors identifies actionable therapeutic targets: a report from the Children’s Oncology Group
Source: Sci Rep. 2020 Jul 23;10:12318. doi: 10.1038/s41598-020-69015-w (PMC7378211; doi:10.1038/s41598-020-69015-w)
Supplement: Supplementary file 1 — Supplementary Information 1. [file 41598_2020_69015_MOESM1_ESM.docx]

Supplementary Data

**Title: Transcriptome analysis of desmoplastic small round cell tumors identifies actionable therapeutic targets: A Report from the Children’s Oncology Group**

**Running Title: RNA-sequencing analysis of DSRCT**

**Authors and affiliations:** Pooja Hingorani^1^, Valentin Dinu^2^, Xiyuan Zhang^3^, Haiyan Lei^3^, Jack F. Shern^3^, Jin Park^2^, Jason Steel^2^, Femina Rauf^2^, David Parham^4^, Julie Gastier-Foster^5^, David Hall^6^, Douglas S. Hawkins^7^, Stephen X. Skapek^8^, Joshua Labaer^2^, Troy McEachron^9, 10^

**Supplemental Figure 1:** Wild type WT-1 is not retained in DSRCT patient samples and JNDSRCT-1 cell line. Sashimi plot of the RNA sequencing data showing the exon usage of WT1 in DSRCT specimens and the JN-DSRCT cell line as indicated by the gray arches. Gray peaks represent RNA sequencing reads. Note that the WT1 locus is in the antisense orientation.

**Supplemental Figure 2:** Specificity of the αWT1 antibody. Immunopreciptiation and western blotting of cell lysates from the JN-DSRCT-1 cells stably expressing an inducible shRNA against WT1. Data is representative of duplicate experiments.

**Supplemental Figure 3:** Expression of both the (-KTS) and (+KTS) variants of *WT1.* (A) RNA-seq coverage plots at the KTS boundary. (B) Quantification of the RNA-seq reads that map to either the (-KTS) or (+KTS) transcripts in the DSRCT patient samples and the JN-DSRCT-1 cell line (labeled “Cell Line”).


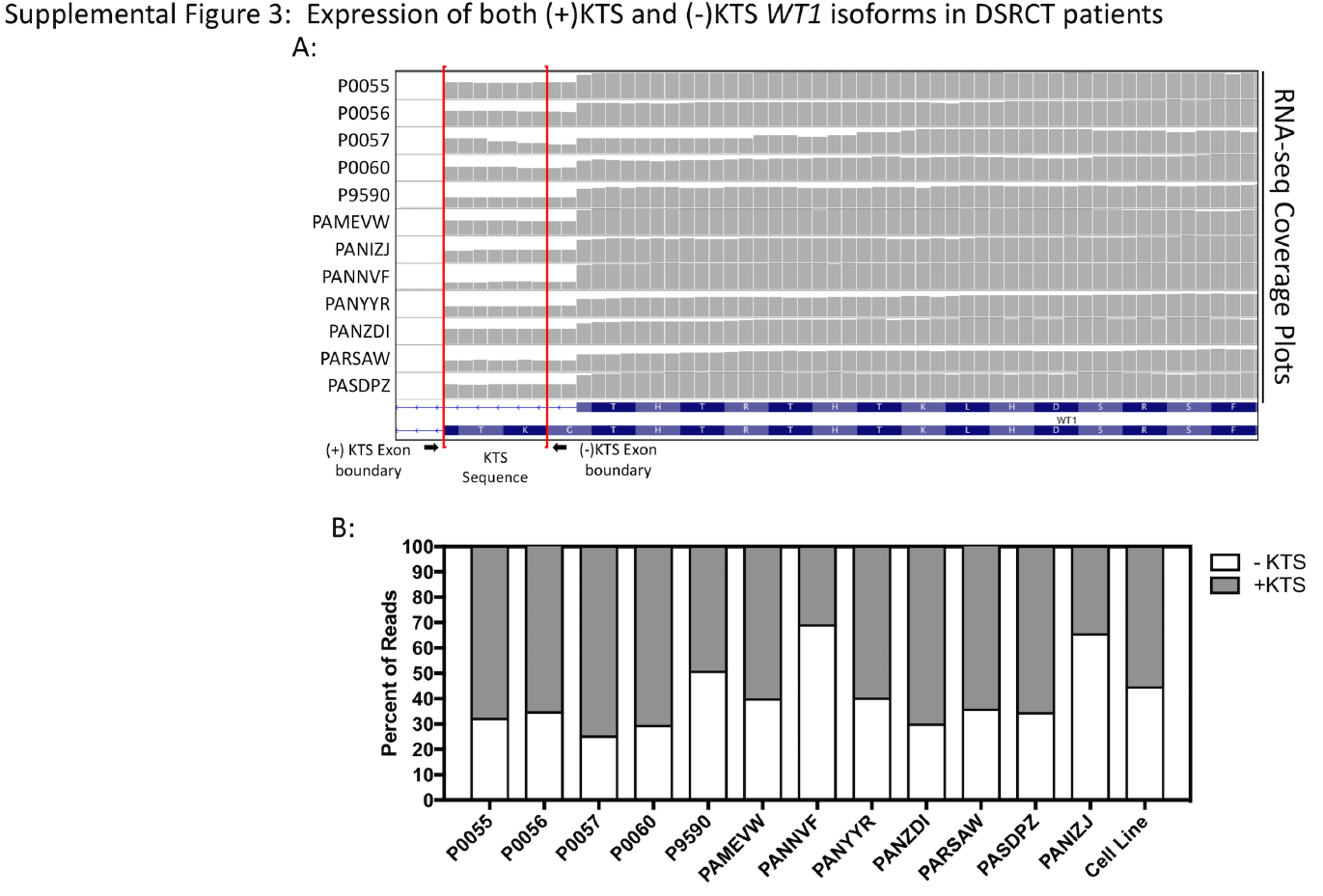


Supplemental Table 1: Primer sequence for EWS-WT1

| **Primer** | **Sequence** | **Reference** |
| --- | --- | --- |
| *EWSR1* exon 7 | 5'-CCAAGTCAATATAGCCAACAG-3' | Lewis TB, *et al.* Mod Pathol. **2007** Mar;**20(3):**397-404 PMID:17334332 |
| *WT1* 3'UTR | 5'-CACCAAATGGCAATGGGCTT-3' | N/A |
|  |  |  |
